# A Narrative Review of Photon-Counting CT and Radiomics in Cardiothoracic Imaging: A Promising Match?

**DOI:** 10.3390/diagnostics15202631

**Published:** 2025-10-18

**Authors:** Salvatore Claudio Fanni, Ilaria Ambrosini, Francesca Pia Caputo, Maria Emanuela Cuibari, Domitilla Deri, Alessio Guarracino, Camilla Guidi, Vincenzo Uggenti, Giancarlo Varanini, Emanuele Neri, Dania Cioni, Mariano Scaglione, Salvatore Masala

**Affiliations:** 1 Department of Translational Research, Academic Radiology, University of Pisa, 56126 Pisa, Italy; 2 Radiology Department of Surgery, Medicine and Pharmacy, University of Sassari, Viale S. Pietro, 07100 Sassari, Italy; 3 Department of Radiology, James Cook University Hospital & Teesside University, Marton Road Marton Rd., Middlesbrough TS4 3BW, UK

**Keywords:** photon-counting computed tomography, radiomics, chest imaging, cardiac imaging, virtual monoenergetic images

## Abstract

Photon-counting computed tomography (PCCT) represents a major technological innovation compared to conventional CT, offering improved spatial resolution, reduced electronic noise, and intrinsic spectral capabilities. These advances open new perspectives for synergy with radiomics, a field that extracts quantitative features from medical images. The ability of PCCT to generate multiple types of datasets, including high-resolution conventional images, iodine maps, and virtual monoenergetic reconstructions, increases the richness of extractable features and potentially enhances radiomics performance. This narrative review investigates the current evidence on the interplay between PCCT and radiomics in cardiothoracic imaging. Phantom studies demonstrate reduced reproducibility between PCCT and conventional CT systems, while intra-scanner repeatability remains high. Nonetheless, PCCT introduces additional complexity, as reconstruction parameters and acquisition settings significantly may affect feature stability. In chest imaging, early studies suggest that PCCT-derived features may improve nodule characterization, but existing machine learning models, such as those applied to interstitial lung disease, may require recalibration to accommodate the new imaging paradigm. In cardiac imaging, PCCT has shown particular promise: radiomic features extracted from myocardial and epicardial tissues can provide additional diagnostic insights, while spectral reconstructions improve plaque characterization. Proof-of-concept studies already suggest that PCCT radiomics can capture myocardial aging patterns and discriminate high-risk coronary plaques. In conclusion, evidence supports a growing synergy between PCCT and radiomics, with applications already emerging in both lung and cardiac imaging. By enhancing the reproducibility and richness of quantitative features, PCCT may significantly broaden the clinical potential of radiomics in computed tomography.

## 1. Introduction

**Figure 1:**
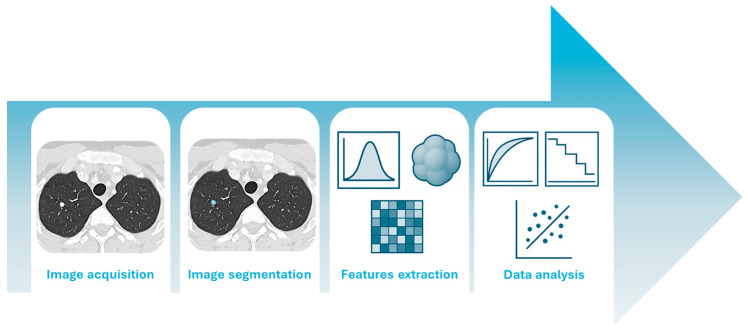
The radiomics pipeline, from image acquisition to data analysis.

Computed tomography (CT) is a widely used X-ray imaging technique known for its high spatial resolution and pivotal role in diagnostic and treatment planning. Conventional CT systems employ energy-integrating detectors (EIDs), which use scintillator materials, such as cesium iodide or gadolinium oxysulfide, to convert X-ray photons (typically in the 30–150 keV range) into visible light. This light is then detected by photodiodes and converted into an analog electrical signal, which is subsequently integrated [1]. As a result, low-energy photons, despite carrying significant contrast information, are underweighted, reducing overall contrast-to-noise performance. EIDs remain limited by reduced tissue contrast, electronic noise, and beam hardening artifacts. Photon-counting CT (PCCT) is a novel technology that overcomes these limitations by using smaller detector elements (pixel sizes down to 0.2 mm) capable of directly converting individual X-ray photons into electrical pulses proportional to their energy, typically in the 20–120 keV range [2,3,4]. This allows for energy discrimination, better use of low-energy photons, and improved spatial resolution. Compared to conventional CT, PCCT provides similar or enhanced image quality, higher iodine contrast-to-noise ratios, lower electronic noise, and fewer beam hardening artifacts [5]. Furthermore, unlike dual-energy CT, which requires dedicated hardware configurations such as dual sources, rapid kVp switching, or dual-layer detectors, PCCT inherently provides energy-resolved data at the detector level. This intrinsic spectral capability allows for the simultaneous acquisition of multiple energy bins from a single scan, enabling advanced post-processing such as the reconstruction of virtual monoenergetic images (VMI) across a wide range of keV levels, and improved tissue characterization without the need for additional radiation exposure or acquisition time [6]. Radiology has played a leading role in driving medical innovation, not only through cutting-edge hardware but also through significant software advancements. While all medical disciplines have benefited from such advancements, the field of radiology has been completely revolutionized. Thanks to the digitization of medical images, it is now possible to extract high-dimensional, mineable data which has led to the emergence of a novel -omics discipline, defined as radiomics [7]. Radiomics refers to the extraction and analysis of large amounts of quantitative imaging features extracted from medical images [8]. A feature can be defined as an attribute or measurable property of the image that mathematically describes specific characteristics. These features vary in complexity, from those describing the mean intensity of individual pixels to those capturing relationships between multiple pixels or regions [7]. The typical radiomics pipeline is outlined in Figure 1.

After acquiring high-quality medical images from patients, radiologists delineate a volume of interest (VOI) that can encompass the lesion or other relevant areas, such as the tumor microenvironment or specific sub-regions [9]. This step, referred to as segmentation, can be performed manually, automatically, or semi-automatically. Radiomic features are then extracted from these VOIs. These features quantify a wide range of imaging characteristics, including intensity, shape, texture, and higher-order statistical patterns. The extracted features are subsequently analyzed (Step IV), beginning with feature selection to reduce dimensionality and prevent overfitting. Model development is then carried out using conventional statistical methods or machine learning (ML) algorithms, a subfield of artificial intelligence (AI) [10,11]. Such high-dimensional quantitative features extracted from medical images capture tissue characteristics that are often imperceptible to the human eye [12,13]. PCCT, with its superior spatial resolution, enhanced contrast-to-noise ratio, and intrinsic spectral capabilities, offers more detailed and accurate image data compared to conventional CT. These improvements can further enhance the quality and reliability of radiomic features, potentially increasing their sensitivity to subtle tissue heterogeneity and improving the performance of predictive models. However, as radiologists must adapt to this new semiology and spatial resolution, also radiomics may be affected by these evolving paradigms. Additionally, radiomic features can be extracted not only from conventional images, acquired at much higher spatial resolution, but also from iodine maps or virtual monoenergetic images (VMI). This substantially increases the number of extractable features and further amplifies the complexity of models relying on such analyses. As such, PCCT may represent a significant technological advancement for radiomics, pushing quantitative imaging beyond the current limits achievable with traditional CT systems. This narrative review investigates the state-of-the-art of radiomics applications in PCCT imaging, with a focus on cardiothoracic diseases.

## 2. Methods

This narrative review was conducted through a comprehensive literature search aimed at identifying studies exploring the application of radiomics applications in PCCT imaging. The search strategy covered the major biomedical databases, i.e., PubMed, Embase, Web of Science, Google Scholar, and Scopus, and included publications up to June 2025. Search terms included “photon-counting detector CT”, “spectral CT”, “radiomics”, “radiomic features”, “texture analysis”, “quantitative imaging”, “precision imaging”, machine learning” and “artificial intelligence”.

We included original research articles, clinical trials, observational studies, and case series involving human subjects, published in English. Conference abstracts, editorials, review papers, and studies not directly addressing PCCT-based radiomic analysis were excluded.

All eligible articles were assessed for relevance, and data were extracted on study design, patient population, imaging protocols (including acquisition and reconstruction parameters), radiomic feature extraction and validation methods, reported clinical endpoints, and acknowledged study limitations. Given the methodological variability across the available literature, a narrative synthesis approach was adopted.

The aim of this review was to provide an evidence-based, clinically oriented overview of current knowledge, highlight the advantages and limitations of PCCT in the radiomics domain, and identify gaps that warrant future investigation.

## 3. Phantom Studies

**Figure 2:**
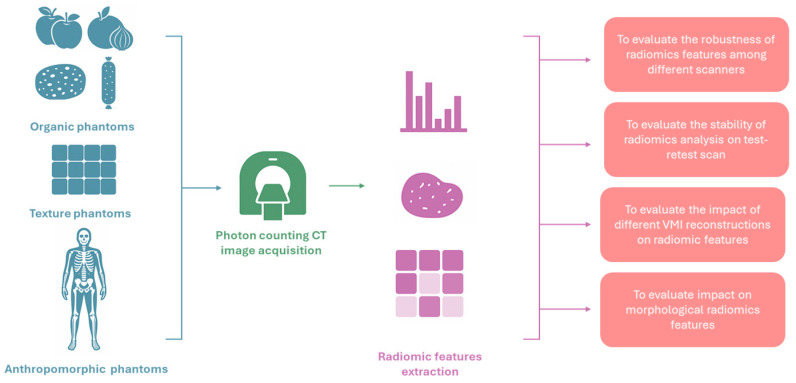
Pipeline of studies investigating the robustness of radiomic features in photon-counting CT images.

Phantom studies play a crucial role in radiologic imaging research by providing controlled conditions for assessing signal variability and the stability of quantitative image features. Numerous investigations have examined how radiomic feature values vary with changes in acquisition parameters and scanner technologies, with the new PCCT now under similar scrutiny (Figure 2).

Different hardware technologies may represent an additional source of variability for such quantitative features. Zhu et al. investigated the robustness of radiomic features among PCCT and dual-energy CT systems and found a 0.157 ± 0.174 (mean ± standard deviation) intraclass correlation coefficient (ICC) for inter-system reproducibility [14].

However, studies focusing solely on PCCT demonstrated that this modality yields highly repeatable radiomic measurements. Hertel et al. reported that almost 70% of extracted features had excellent test–retest stability (concordance correlation coefficients > 0.9) across repeated PCCT scans of organic phantoms [15]. Moreover, Wolf et al. investigated the impact of different VMI reconstructions on radiomic features reproducibility. As a result, out of 93 radiomic features, 36 (38.7%), 28 (30.1%), and 33 (35.5%) were not significantly different in apples, oranges, and onions, respectively [16]. Similarly, Dawi et al. demonstrated that things may get a little bit complicated if factors such as VMI energy, reconstruction kernel, and matrix size are taken into account [17]. Such reconstruction parameters can markedly affect feature stability extracted from two organic phantoms, i.e., a natural wet sponge and a dry sausage. However, the authors managed to identify an optimal reconstruction configuration, achieving about 90 out of 93 features classified as stable [17].

Zhang et al. investigated the impact of such acquisition parameters on the robustness of radiomics in a phantom with 28 texture materials. Radiomic features demonstrated high robustness to variations in tube voltage, radiation dose, reconstruction strength, and convolution kernel, indicating that these quantitative metrics remain reproducible under a broad range of routine imaging conditions. Still, the features proved brittle when high-pitch acquisitions or increased slice thickness were employed. High-pitch helical scans can introduce motion-related artifacts and reduce temporal sampling, while thicker slices exacerbate partial volume effects and diminish spatial resolution; both factors compromise the depiction of fine tissue heterogeneity, resulting in significant variability of texture-based features [18]. Overall, these phantom-based analyses indicate that PCCT can deliver very consistent radiomic measurements under carefully controlled conditions.

However, radiomic features stability does not automatically translate to more accurate quantification of lesion characteristics compared to conventional CT. To investigate such point, Sharma et al. demonstrated in an anthropomorphic thoracic phantom that PCCT significantly reduced the error in radiomic feature estimation across multiple lesion types and sizes when compared with conventional CT, highlighting its potential to improve diagnostic fidelity in radiomics applications [19].

## 4. Chest Imaging

The lung is an organ with inherently high contrast resolution compared to other organs, due to interfaces between tissues that have significantly different X-ray attenuation. Therefore, optimizing lung CT imaging primarily depends on achieving high spatial resolution, especially important for assessing fine and subpleural abnormalities at the lung–pleura interface, such as those seen in interstitial lung diseases (ILD), as well as sufficient temporal resolution to reduce respiratory and cardiac motion artifacts [20,21,22]. PCCT offers significant advantages as its inherently high spatial resolution markedly improves the visualization of fine parenchymal structures and peripheral airways, which is particularly valuable in the early identification of subtle ILD features or in evaluating structural changes following treatment or surgery [20]. The substantial reduction in electronic noise and image artifacts, such as the suppression of blooming artifacts from surgical clips, along with higher spatial resolution, enables earlier and more accurate detection of post-surgical pathological changes, such as bronchial wall thickening [21]. Furthermore, the inherently higher signal-to-noise ratio (SNR) of PCCT results in better assessment of ILD, where subtle changes in ground-glass opacity or septal thickening, especially on follow-up scans, can greatly influence clinical decisions [23]. PCCT offers the possibility to generate VMI and iodine maps from every scan without added acquisition time and without increasing radiation exposure. Baseline (virtual non-contrast, VNC) images can also be obtained. These characteristics open promising opportunities in thoracic imaging, for instance, in Computed Tomography Pulmonary Angiogram (CTPA) for pulmonary embolism, and pave the way for spectral radiomics in assessing pulmonary, pleural, and mediastinal lesions [20,24,25]. Importantly, PCCT can maintain high image quality at extremely low radiation doses. The chest is a region where dose reduction is particularly important due to frequent imaging in oncology patients, long-term ILD follow-up (often starting at a young age), and lung cancer screening programs. PCCT allows for high-resolution diagnostic imaging with radiation doses below 1 mSv, while maintaining volumetric accuracy and structural detail [23,26]. Overall, these benefits not only enhance the quality and reliability of thoracic imaging in clinical practice but also lay a strong foundation for advancing high-quality radiomics research and applications. The potential synergy between PCCT and radiomics has already been the subject of investigation in some proof-of-concept studies in both lung nodule and ILD clinical setting. A simulation study was conducted by Sharma et al. to evaluate the accuracy of morphological radiomic features of lung nodules using silicon-based photon-counting CT (Si-PCCT) compared to conventional CT [19]. Using anthropomorphic phantoms with synthetic lung lesions, the authors tested multiple imaging conditions. Si-PCCT significantly reduced the mean relative error in shape feature estimation, particularly for features independent of other metrics, compared to energy-integrating CT. The greatest improvements were associated with enhanced in-plane spatial resolution. These findings suggest that Si-PCCT provides quantitative advantages for radiomics, especially in morphology-focused applications. Similarly, McCabe et al. highlighted how PCCT may enhance the reproducibility of radiomic features by reducing variability linked to image acquisition parameters [27]. Among these, slice thickness had the greatest influence, while dose and reconstruction kernel had minimal impact. PCCT demonstrated higher consistency than conventional CT in detecting detailed morphological traits like spiculation. These results support the role of PCCT in improving radiomics-based lung nodule characterization.

In the ILD clinical setting, Koo et al. investigated the impact of PCCT on the performance of a quantitative ML model compared with conventional CT [28]. The authors reported that quantitative features extracted from PCCT, and conventional CT were highly similar and largely consistent. However, PCCT allowed better delineation of honeycombing and showed a stronger correlation with pulmonary function tests. In contrast, the correlation between ground-glass extent and pulmonary function tests decreased when using PCCT. These findings suggest that the introduction of advanced technologies such as PCCT may require model adjustments to optimize performance.

## 5. Cardiac Imaging

PCCT represented a major innovation in cardiac imaging for its higher spatial resolution, reduced image noise, and inherent spectral capabilities when compared to conventional EID-CT system [29]. These advantages translate, in a better visualization of coronary arteries, into a more accurate delineation of atherosclerotic plaque morphology and improved myocardial tissue characterization, especially through VMI reconstruction that could better enhance contrast in either calcified or non-calcified structures [30,31]. The improved anatomical and functional imaging quality could provide a fertile substrate for radiomics. Some proof-of-concept studies have explored the integration of radiomics with PCCT for cardiac applications. First of all, Kravchenko et al. investigated the intra-individual stability and reproducibility of pericoronary adipose tissue (PCAT) radiomic features between PCCT and conventional EID-CT in patients undergoing clinically indicated coronary CT angiography on both systems [30]. Image acquisition parameters were standardized, and PCCT datasets were reconstructed both at a down-sampled 0.6 mm slice thickness (PCCT-DS) to match the EID-CT scan and at 0.2 mm in ultrahigh-resolution mode (PCCT-UHR). A total of 110 radiomic feature classes were extracted. Feature stability was high between EID-CT and PCCT-DS (91%), but markedly lower when ultrahigh-resolution datasets were included (EID-CT vs. PCCT-UHR: 55%; PCCT-DS vs. PCCT-UHR: 51%). Inter-scanner reproducibility was poor in both comparisons (EID-CT vs. PCCT-DS, median ICC: 0.43 (0.03–0.69); EID-CT vs. PCCT-UHR, median ICC: 0.29 (0.01–0.51)). Conversely, reproducibility improved substantially within PCCT datasets (PCCT-DS vs. PCCT-UHR, ICC: 0.72 (0.48–0.83)), despite differences in slice thickness. These results suggest that radiomic features derived from PCCT exhibit greater internal consistency compared with cross-scanner reproducibility. However, when dealing with the complexity of PCCT, several variables potentially affecting radiomic features reproducibility should be taken into account. Wolf et al. explored the potential impact of PCCT on radiomics features stability not only in vitro, as above discussed, but also in vivo, on 23 patients who had undergone coronary computed tomography angiography on a first generation PCCT system [16]. Patients’ left ventricular myocardium was segmented for all VMI and 93 radiomic features were extracted. For the left ventricular myocardium, the proportion of stable features was high at VMI levels ≥ 90 keV (90 vs. 120 keV, 77.4%; 90 vs. 190 keV, 83.9%; 120 vs. 190 keV, 89.3%), whereas comparisons with lower VMI levels resulted in substantially fewer reproducible features (40 vs. 55 keV, 8.6%). Another different type of reconstruction, i.e., a potential source of variability, was investigated by Canalini et al. The authors investigated the potential impact of virtual-non-contrast (VNC) reconstruction of radiomics features, compared to radiomics features extracted from true non-contrast (TNC) images [32]. TNC and VNC series from 84 patients were retrospectively analyzed. In each case, the myocardium and epicardial adipose tissue (EAT) were semi-automatically segmented on both reconstructions, and 105 radiomics features were extracted. In the myocardium, 41 stable features were identified, while in the epicardial fat 40 stable features were found, with the three highest-ranked belonging to first-order statistics. Overall, 24 features (22.8%; 24/105) demonstrated stability in both anatomical structures. Notably, significant differences in the correlation of radiomics features between VNC and TNC volumes of the myocardium and EAT suggested that the two reconstructions may differ more than initially assumed, indicating that they may not be interchangeable and that such differences could have clinical implications. However, a study by Cui et al. demonstrated that PureCalcium-based VNC (VNCPC) may improve such reproducibility [31]. The authors investigated the reproducibility of CT radiomic features of EAT in 52 patients undergoing PCCT angiography. EAT volume (EATV) and density (EATD) were quantified on TNC, VNCPC, and conventional VNC (VNCConv) reconstructions. Both VNCPC and VNCConv tended to underestimate EATV and overestimate EATD, but VNCPC demonstrated higher correlation and agreement with TNC than VNCConv. Importantly, all categories of RFs derived from VNCPC showed greater reproducibility compared with VNCConv. These results suggest that VNCPC may serve as a reliable substitute for TNC in the assessment of EAT, exceeding the performance of VNCConv. Owing to the higher spatial resolution and improved signal-to-noise ratio, PCCT may allow to better capture subtle myocardial texture alterations related to coronary artery calcification. In the study by Ayx et al., 30 patients were retrospectively analyzed and divided into two subgroups according to the severity of coronary artery calcification (Agatston score 0 vs. ≥100) [33]. Using random forest feature selection, a subset of four higher-order radiomics features was identified that discriminated myocardial texture between the two groups. These findings indicate that texture alterations of the left ventricular myocardium are associated with the severity of coronary artery calcification as estimated by the Agatston score. Radiomics-based myocardial characterization on PCCT images may provide further insights, as demonstrated by Hertel et al. The authors conducted a retrospective single-center study including 90 patients to evaluate whether radiomic features extracted from semi-automatic segmentation of the left ventricular myocardium on PCCT could differentiate younger (<60 years) and older (>70 years) individuals, potentially serving as imaging biomarkers of myocardial aging [34]. EAT density and thickness were also assessed but showed no significant differences between age groups. A random forest model achieved moderate accuracy (0.74); among the extracted features, “wavelet-HLH_glszm_GrayLevelNonUniformity” was identified as most relevant, indicating greater myocardial heterogeneity in younger patients and relative homogeneity with aging, possibly reflecting diffuse fibrotic remodeling. Overall, the study demonstrated that PCCT-derived radiomic texture features may differentiate myocardial aging patterns more effectively than conventional EAT metrics, supporting their potential role as non-invasive imaging biomarkers for personalized cardiovascular risk stratification. Beyond myocardial characterization, radiomics can also be applied to PCCT for the direct assessment of coronary plaques. In this context, Dunning et al. demonstrated that radiomics-based ML applied to PCCT could differentiate low-risk from high-risk coronary plaques [35]. In their study, 25 plaques were segmented from 19 patients undergoing coronary CTA with PCCT. Five types of reconstructions were generated, including virtual VMIs at 50, 70, and 100 keV, iodine maps, and VNC images reconstructed using an iterative algorithm (QIR), a quantitative kernel (Qr40), and 0.6-mm/0.3-mm slice thickness/increment. A set of statistically significant features was extracted for each dataset (18 at 50 keV, 32 at 70 keV, 43 at 100 keV, 16 from iodine maps, and 55 from VNC) and subsequently classified according to accuracy. Among the reconstructions, 100 keV VMIs and VNC images achieved the best performance, likely due to reduced blooming artifacts from iodine and calcium. Overall, this study demonstrated that PCCT combined with radiomics and ML can accurately classify high-risk coronary plaques, potentially enabling the identification of vulnerable plaques beyond conventional CT and thereby improving non-invasive cardiac risk stratification.

## 6. Discussion

This narrative review described the initial evidence regarding PCCT and the potential to substantially affect software-driven domains of image analysis, particularly radiomics and ML. Early phantom-based investigations demonstrated that radiomic features extracted from PCCT are subject to variability when compared with EID-CT [14], while intra-scanner stability was generally optimal [15,30]. However, PCCT introduces a significantly higher degree of complexity, with multiple acquisition and reconstruction variables influencing feature behavior. PCCT represents a technological step forward, more resembling the complexity of magnetic resonance imaging, where the need for standardized protocols is essential to ensure reproducibility. Indeed, the capability of PCCT to reconstruct VM and VNC datasets exponentially increases the amount of information that can be extracted from a single examination, further expanding the radiomic feature dimensionality.

In thoracic imaging, preliminary investigations suggest that PCCT can enhance the reproducibility and accuracy of quantitative image analysis, especially in shape feature estimation of lung nodules [19]. However, such improvement also come with a need for model recalibration, as shown by Koo et al. in the ILD setting [28].

Collectively, these studies underline the potential of PCCT to improve feature stability and clinical correlations, but they also emphasize the challenges of integrating PCCT into radiomics workflows without appropriate model adjustments.

In the cardiac imaging field, the interplay between PCCT and radiomics appears even more promising, but equally complex. Radiomics feature stability was demonstrated highly dependent on VMI energy level, with improved reproducibility at higher keV values [16]. Reconstruction techniques represent another source of variability, as significant differences were identified in features derived from VNC versus TNC images, with limiting interchangeability [32]. Interestingly, VNCPC approach improved radiomics features reproducibility of epicardial adipose tissue features, suggesting a possible technical solution [31]. Beyond reproducibility, PCCT-based radiomics also shows promise in clinical applications. Myocardial texture features extracted from PCCT correlated with the severity of coronary artery calcification, potentially reflecting subtle tissue remodeling, and also allowed differentiation of myocardial aging patterns more effectively than conventional epicardial fat metrics, with wavelet-based features emerging as candidate imaging biomarkers [33,34]. Beyond these early observations, a more advanced level of myocardial tissue characterization could have direct procedural implications. Integration of CT-derived fibrosis mapping into electrophysiological workflows has already proven useful for ventricular tachycardia ablation planning, where detailed anatomical assessment helps refine patient selection, optimize ablation targeting, and improve outcome prediction [36]. Such examples highlight the translational potential of PCCT-radiomics integration to guide revascularization and electrophysiological strategies, moving from image-based quantification toward patient-specific therapeutic planning.

Finally, PCCT-derived radiomics combined with machine learning has been demonstrated able to discriminate between low-risk and high-risk coronary plaques, with reconstructions at 100 keV and VNC images achieving the best performance due to reduced blooming artifacts [35]. Taken together, the current body of evidence highlights both opportunities and challenges. PCCT offers superior spatial resolution, enhanced signal-to-noise ratio, and intrinsic spectral capabilities that may improve the accuracy, reproducibility, and clinical value of radiomic features. At the same time, the increased complexity of acquisition and reconstruction parameters introduces new variability factors that require harmonization and standardization. Lessons learned from MRI, where consensus protocols are critical to ensure cross-scanner reproducibility, may serve as a model for developing similar strategies in PCCT. Importantly, the clinical studies conducted to date are small and heterogeneous, reinforcing the need for multicenter trials and larger patient cohorts to validate findings, explore spectral radiomics applications, and test their integration into machine learning pipelines [37,38,39,40,41,42,43]. Ultimately, PCCT represents a promising but challenging frontier for radiomics in cardiothoracic imaging. Its ability to generate richer, multi-parametric datasets aligns well with the needs of artificial intelligence and precision medicine, but careful methodological development, standardization, and validation are essential prerequisites for its translation into robust clinical practice.

## Data Availability

No new data were created or analyzed in this study. Data sharing is not applicable to this article.
